# Using Different Cage Enrichments to Improve Rabbits’ Performance, Behavior, and Welfare

**DOI:** 10.3390/ani14152271

**Published:** 2024-08-04

**Authors:** Menna Elsayed, Farid Soliman, Osama Elghalid, Karim El-Sabrout

**Affiliations:** Poultry Production Department, Faculty of Agriculture, Alexandria University, Alexandria 21545, Egypt

**Keywords:** behavioral activities, carcass quality, cecum bacterial count, corticosterone, environmental enrichment, growth, housing, immunity

## Abstract

**Simple Summary:**

Environmental stressors refer to the various factors in the surrounding environment that impose serious threats to animal welfare and production. They can increase susceptibility to behavioral issues and infections, as well as reduce growth rates and reproductive performance. Conventional housing systems represent one of the critical environmental stressors that have a significant negative impact on rabbits’ well-being and productivity. Therefore, enriching rabbit cages helps to reduce stress and promote natural behaviors, enhancing the health and productivity of rabbits.

**Abstract:**

Environmental enrichment is about improving the surroundings in which your animal lives by providing opportunities to express behavioral activity normally, which in turn has a great impact on the animal’s welfare and productivity. The aim of the present study is to investigate the impact of using different enrichment cage tools (a rubber floor, plastic-colored balls, and a mirror) on rabbits’ physiology, productivity, carcass quality, behavior, and welfare. A total of 84 weaned rabbits (V-line) were randomly and equally assigned to 4 groups, each with 7 replicates (3 rabbits/replicate). The 1st rabbit group (T1) served as a control, while the 2nd group (T2) was enriched with rubber floors. The 3rd group (T3) was enriched with plastic-colored balls, and the 4th group (T4) was enriched with mirrors. Productive traits, including the weekly body weight and feed intake, as well as the carcass characteristics, were measured. Hematological parameters and biochemical constituents were determined according to the reference’s description. Furthermore, behavioral activities, such as walking, resting, feeding, and drinking, were observed. According to the results, enriching the rabbit cages with plastic-colored balls and mirrors improved the marketing body weight and feed conversion rate. It also improved carcass quality characteristics, such as the carcass weight and dressing percentage. The T3 and T4 rabbits had higher RBCS, Hb, and hematocrit levels as well as lower WBCS levels. They also had significantly higher total protein, globulin, glucose, AST, and IgG values than other treatments. In addition, they had significantly lower corticosterone levels and fear responses. Therefore, it is recommended to use plastic-colored balls and mirrors for rabbit farming for better productivity, behavior, and welfare.

## 1. Introduction

Rabbits (*Oryctolagus cuniculus*) are well-known sensitive, active, energetic, inquisitive, and social animals. In intensive/commercial rabbit-production systems, animals are typically housed in wire mesh cages. These systems may cause stress and harm to rabbits. The welfare of farmed rabbits is primarily determined by their housing conditions. Thus, seeking better housing conditions that fit animal welfare aspects is critical [1]. 

Rabbits’ physiological and behavioral performance are influenced by their surroundings, particularly their housing conditions. Practical management and husbandry solutions are required to improve rabbits’ behavior, production, and well-being [2,3]. From this viewpoint, providing environmental enrichments that increase environmental diversity can improve the rabbit’s quality of life while lowering stress. Environmental enrichment promotes the mental and physical well-being of caged animals by addressing species-specific requirements [4,5]. It is also necessary for animal production, since it can reduce abnormal behavior while increasing animal comfort [6,7]. Additionally, environmental enrichment adds details and opportunities that allow animals to make decisions that improve their own quality of life [8,9]. 

Several previous studies have investigated the use of certain tools and equipment to enrich animal cages. Hansen and Berthelsen [2] found that rabbits kept in an enriched cage, with an accessible shelter and a high stage, had better welfare than rabbits kept in a conventional cage system, because they had a better chance of interacting with the environment. According to Princz et al. [10], the presence of gnawing sticks in rabbit cages altered some behavioral activities of rabbits, such as resting, locomotive, and aggressive behaviors, while decreasing the frequency of physical injuries. Dalle Zotte et al. [11] observed that providing rabbit cages with mirrors enhanced rabbit behavior and welfare. Furthermore, Rashed and El-Edel [12] revealed that replacing the wire net floor with plastic can improve rabbit welfare and production. Working on different enrichment cages, Feng et al. [13] discovered that rabbits raised in cages enriched with cans of beans had better behavioral activities and cecal microbiota compositions compared to rabbits in conventional cages. Additionally, Musco et al. [14] found that enriched rabbits exhibited higher carcass weights than a control group. 

Although there are many tools for enriching an animal’s environment, and many of these have been studied on certain small livestock, such as poultry, their application to rabbits remains limited. Hence, this study aims to investigate the effects of using different enrichment cage tools (a rubber floor, colored balls, and a mirror) on rabbits’ productivity, physiological traits, carcass quality, behavior, and welfare.

## 2. Materials and Methods

### 2.1. Animal Ethics

The experimental procedure was performed in accordance with the guidelines of the Experimental Animal Care Committee Ethics of Alexandria University (Alex. Agri. 092401101).

### 2.2. Experimental Design

**Figure 1:**
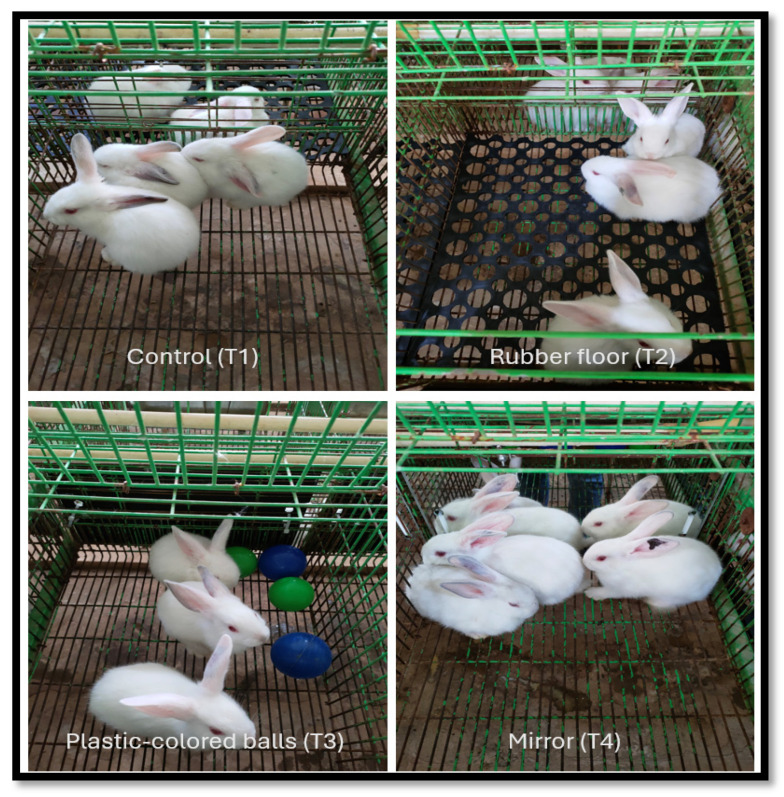
Different cage enrichments to improve rabbits’ performance, behavior, and welfare.

The experiment was conducted in the winter (December–January) of 2023–2024 at the poultry research center of Alexandria University, Alexandria (31.2001° N, 29.9187° E). A total of 84 weaned rabbits “V-line” [15], at 28 days of age, were randomly and equally assigned to 4 treatments (Figure 1), each with 7 replicates (3 rabbits per replicate). The 1st rabbit group (T1) served as a control (wire-net floor), while the 2nd group (T2) was enriched with a black rubber floor. The 3rd group (T3) was enriched with plastic-colored balls (blue and green, as rabbits can identify these colors), and the 4th group (T4) was enriched with a mirror fixed to one side of the cage. The experiment lasts for 6 weeks. The productive traits, including the weekly body weight and feed intake, and carcass characteristics were measured. Hematological parameters and biochemical constituents were determined according to the reference’s description. Furthermore, behavioral activities, such as walking, resting, feeding, and drinking, were observed. 

### 2.3. Animal Husbandry

In an open-system building, the growing rabbits were housed collectively (3 rabbits/cage) in one-level wire cages (50 × 40 × 30 cm each). They were managed under similar environmental conditions (average temperature, 21 °C; humidity rate, 60%; good ventilation; out of direct sunlight). They were given ad libitum access to a commercial pelleted diet (containing around 18% crude protein, 12% crude fiber, and 2650 kcal/kg of metabolizable energy) and fresh clean water during the fattening period. All the rabbits were kept under the same managerial, environmental, and hygienic conditions.

### 2.4. Data Collection

#### 2.4.1. Productive Performance 

The body weight, body weight gain, feed intake, feed conversion rate, and mortality rate of rabbits were recorded weekly during the experiment.

#### 2.4.2. Hematological and Biochemical Parameters

At the end of the experiment, 7 rabbits from each group were chosen at random to collect blood samples during slaughter for hematological and biochemical analyses. The count of red blood cells (RBCs) was determined on an acridine orange (AO) bright-line hemocytometer using a light microscope at 400× magnification, while the count of white blood cells (WBCs) and their differentiation were determined according to El-Saadany et al. [16] and Altan et al. [17]. The hemoglobin (Hb) was determined by the cyanmethemoglobin method, and the hematocrit (%) was measured using Wintrobe hematocrit tubes [18].

The total protein concentration (g/dL) was measured according to Henry et al. [19], and the albumin concentration (g/dL) was estimated using Doumas et al.’s [20] method, while the globulin concentration (g/dL) was calculated by subtracting the total protein from the albumin. The activity of liver enzymes (aspartate aminotransferase (AST) and alanine aminotransferase (ALT)) was assayed in plasma by Reitman and Frankel’s [21] method using a specific kit (Diamond Diagnostics Chemical Company, Cairo, Egypt). The plasma glucose concentration was estimated according to Trinder [22] using the instructions of a specific kit (Diamond Diagnostic Company, Cairo, Egypt). Creatinine was evaluated according to Schirmeister et al. [23]. Plasma Triiodothyronine (T_3_) levels were analyzed using radioimmunoassay (RIA) kits (Diagnostic Systems Laboratories, USA, Webster, TX, USA) using the method published by Hollander and Shenkman [24]. The plasma corticosterone concentration was measured using the Corticosterone Competitive ELISA kit (Bioassay Technology Laboratory, Shanghai, China), as described by the manufacturer. The plasma Immunoglobulin G (IgG) concentration was determined using the IgG ELISA kit (Enzyme-linked Immunosorbent Assay kit, Cloud-Clone Corp., Katy, TX, USA), while the Immunoglobulin M (IgM) was assessed using the IgM ELISA kit (Immunology Consultants Laboratory, Inc., Portland, Oregon, USA), according to the manufacturer’s instructions. The total cholesterol (TC) levels, triglycerides (TGs), and concentrations of HDL (high-density lipoprotein) and LDL (low-density lipoprotein) were determined according to Lacková et al. [25].

#### 2.4.3. Carcass Quality 

The rabbits, at the end of the experiment, were slaughtered three hours after feed removal. Seven rabbits from each group were chosen at random to determine the carcass quality, including the carcass weight, dressing percentage, intestinal length, and internal organ relative weights.

#### 2.4.4. Cecal Microbiota 

Cecum microbial samples were collected from 7 rabbits per group at the end of the experiment (after slaughtering). *Lactobacillus plantarum*, *Salmonella enteritidis*, and *Escherichia coli* were counted as described by El-Shafei et al. [26].

#### 2.4.5. Behavioral Activities

The behavioral activities of the rabbits were observed directly by an experimenter (outside of the view of the tested rabbits), from day 1 of the experiment (at 28 days of age) until the end of the experiment (at 70 days of age), and by using digital video cameras connected to a monitor. The frequency (or event) direct observing or recording in this study is a way to measure the number of times a behavior occurs within 6 h per day (from 7 a.m. to 1 p.m.). The experimenter’s observations and the video recording were then analyzed to assess different behavioral acts: walking, running, standing, resting, eating, drinking, smelling, self-grooming, urination, fighting, and fear response. An observed severe behavior was scored (+1), and a mild behavior was scored (−1), compared to the rabbits’ normal behavior (0) inside the rabbitry. The studied behavioral activities of the rabbits are presented in Table 1.

#### 2.4.6. Statistical Analysis 

Data were statistically analyzed using the General Linear Model (GLM) procedure in accordance with the SAS program (Version 15.1 2018, North Carolina, NC, USA). All values are presented as means with a standard error of the mean. Significant differences among treatments were subjected to Tukey’s test. Results were considered significant at *p* ≤ 0.05. 

## 3. Results

According to the results in Table 2, enriching the rabbit cages with plastic-colored balls and mirrors positively (*p* ≤ 0.05) affected the rabbits’ productive performance. The T3 and T4 rabbits had the best feed conversion rate compared to other treatments, while the T3 rabbits had the highest (*p* = 0.001) final body weight (marketing weight) (4.17%) and body weight gain (5.82%). There were no significant (*p* > 0.05) differences in the feed intake and mortality percentage among the treatments.

According to the results in Table 3, enriching the rabbit cages impacted the blood biochemical parameters of the rabbits. The T3 and T4 rabbits had significantly (*p* ≤ 0.05) higher total protein, globulin, glucose, AST, and IgG values than other treatments. They had significantly (*p* = 0.004) lower corticosterone levels. The T3 rabbits had the highest (*p* ≤ 0.05) albumin, IgM, and T_3_ values, while the T4 rabbits had the highest cholesterol, triglycerides, and HDL values.

The results in Table 4 show that enriching the rabbit cages influenced the blood hematological parameters of the rabbits. In comparison to other treatments, the T3 and T4 rabbits had higher (*p* ≤ 0.05) RBC, Hb, and hematocrit levels as well as lower (*p* ≤ 0.028) WBC levels. There were no significant (*p* > 0.05) differences in the differential WBCs.

Table 5 represents the effects of enriching the rabbit cages with respect to carcass characteristics and the relative weight of the internal organs of rabbits. According to the results, the T3 and T4 rabbits had a greater (*p* ≤ 0.05) carcass weight, dressing percentage, and intestinal length compared to other treatments. There were no significant (*p* > 0.05) differences in the liver, heart, kidney, and spleen percentage relative weights.

According to the results in Table 6, enriching the rabbit cages with rubber floors, plastic-colored balls, and mirrors had no significant (*p* > 0.05) impact on the cecal bacteria counts of the growing rabbits. 

The effects of different enrichment cages on the behavioral activities of the rabbits are presented in Table 7. The results indicate that enriching the rabbit cages affected the behavioral activities of the rabbits. The T3 and T4 rabbits showed lower standing and fear response values, while exhibiting higher resting and normal self-grooming values.

As a general result, enriching the rabbit cages with plastic-colored balls and mirrors had positive effects on the rabbits’ productivity, carcass quality, behavior, and welfare. Using enriched cages improved the marketing body weight and feed conversion rate. It also improved the carcass quality characteristics, such as the carcass weight and dressing percentage. Moreover, it decreased the fear response as well as enhanced the rabbits’ health and immunity.

## 4. Discussion

Animal welfare has recently gained interest, especially in developing countries. Environmental stress on animals could be due to traditional/poor housing conditions [27,28]. Standard-sized wire cages, as a conventional cage housing system, are often used to house growing/fattening rabbits in groups of three or more; therefore, they may have a negative impact on the well-being and productivity of rabbits. Environmental enrichment is intended to help rabbits deal with their surroundings and reduce stress. Various safe and inexpensive objects, tools, or toys, which are commercially available, can enrich rabbit cages. The current research was performed to study the effects of employing a rubber floor, colored balls, and a mirror, in different environmentally enriched cages, on the physiological status, productive performance, behavior, and welfare of growing rabbits.

Based on the data presented in Table 2, enriching the rabbit cages with plastic-colored balls (T3) and mirrors (T4) significantly increased the rabbits’ productive performance, such as the final body weight and body weight gain. Furthermore, the T3 and T4 rabbits had the best feed conversion rate with no significant differences in the feed intake among the treatments. These findings may be due to improving certain behaviors of the rabbits, such as resting, and boosting some biological functions in rabbit bodies, such as the metabolism, as well as reducing stress levels, which can alter gene expressions and impact critical physiological processes, like growth, as a result of using these enrichment objects. Similarly, enriching the rabbits’ environment has been shown to improve rabbit husbandry and well-being, which has the largest effect on productivity [5,11,13,29]. Rashed and El-Edel [12] assessed the effects of different floor types (plastic, wire, or combination) on the behavior, welfare, and productivity of growing rabbits. They found that the plastic floor, either alone or combined with wire, significantly increased exploration, walking, and body-care behaviors compared to those reared on the wire. Furthermore, the body weight and daily weight gain for a group reared on plastic floors were significantly higher than those on wire or combination floors. Similarly, Gharib et al. [30] noticed that placing plastic or rubber mats on the floor of a wire cage reduced aggressive and abnormal behaviors, while lowering cortisol levels and improving growing rabbits’ productivity and welfare. The aforementioned results are also aligned with the findings of Trocino et al. [7], who evaluated the use of two types of enrichment (an elevated plastic-slatted platform and/or a plastic hiding tube) on the behavioral activities and productive performance of growing rabbits housed in collective pens within large groups. They found that using an elevated plastic-slatted platform allows rabbits to enhance their behavioral range and movement choices without adverse effects on production traits.

According to the results in Table 3, enriching the rabbit cages improved some blood biochemical parameters of the rabbits. The T3 and T4 rabbits had significantly higher total protein, globulin, glucose, AST, and IgG values than those of other treatments. They also had significantly lower corticosterone levels. Furthermore, the T3 rabbits had the highest albumin, IgM, and T_3_ values, while the T4 rabbits had the highest cholesterol, triglyceride, and HDL values. These results are in agreement with Musco et al. [14] and Liang et al. [31], who indicated that environmental enrichments could impact certain blood biochemical indices of rabbits. Working on chickens, Son et al. [32] and Zahoor et al. [33] found that enriching animal environments altered the blood total protein, cholesterol, glucose, creatinine, and corticosterone levels. Alterations in certain blood biochemical parameters could have been attributed to changes in the hypothalamus–pituitary threshold level [34]. Several previous studies have also indicated that environmental enrichments can impact animals’ physiological responses, including their catalytic activity and respiration, leading to an improved overall physiological performance [13,14,35]. In the current study, we found that enriching rabbit cages with plastic-colored balls increased the total protein, globulin, albumin, IgM, and T_3_ levels and lowered the corticosterone levels, which positively affected the growth and health of the rabbits. However, this increase can be explained by how the animal interacts with the objects in its surroundings while changing the rhythm of life and daily vital behavioral activities, which has a great impact on its well-being, ultimately affecting its production. 

The results in Table 4 show that enriching rabbit cages can also influence certain hematological parameters of rabbits within the normal range. The T3 and T4 rabbits had higher RBC_S_, Hb, and hematocrit levels and lower WBC levels compared to other treatments. It is well-known that blood biochemical and hematological analyses can reflect animal health and production [16,36]. The blood picture monitors metabolic changes and provides an overall picture of the animal’s physiological activities [37,38]. According to the results of the current study, enriching rabbit cages with plastic-colored balls and mirrors can successfully recover most of the blood biochemical and hematological parameters to normal ranges, resulting in the better health and productivity of rabbits. 

Table 5 represents the effects of enriching the rabbit cages on the carcass characteristics and the relative weight of the internal organs in the rabbits. From the results, the T3 and T4 rabbits had greater carcass weight, dressing percentage, and intestinal length values compared to other treatments. There were no significant differences in the liver, heart, kidney, and spleen percentage relative weights. The improvement that occurred in the carcass traits can be due to the enhancement of blood biochemical and hematological indices, in addition to improving the overall performance and welfare of the rabbits [39]. Furthermore, an increasing intestine length, which may be an indirect reflection of the enriched material stimulation or a result of increasing Insulin-like growth factor 1(IGF-1) levels in enriched rabbits [13], had a substantial influence on enhancing the feed conversion rates and body weights [40]. Musco et al. [14] reported that mirrors can represent a valid solution by improving the rabbit’s welfare and, at the same time, ensuring good growth performance and carcass quality traits. They revealed that rabbits raised in cages enriched with mirrors showed the best feed conversion rate and dressing percentage. Similarly, Feng et al. [41] found that enriched rabbits exhibited higher carcass weights than a control group. The use of mirrors can improve a rabbit’s growth performance and carcass traits by lowering adverse behavioral activities, such as fear responses. Working on a different species, Zahoor et al. [33] conducted an experiment to investigate the effects of using colored balls on the performance of broiler chickens. They found that the addition of colored balls encourages birds to engage in physical activity and enhances carcass quality.

According to the results in Table 6, enriching the rabbit cages with rubber floors, plastic-colored balls, and mirrors had no significant impact on the cecal bacteria counts of the growing rabbits. On the other hand, Feng et al. [13] observed that the composition and diversity of cecal microbiota were affected by cages enriched with cans of beans. Certain differences in the findings could be attributed to employing different enrichment tools, the rabbits’ age, and surrounding environments. 

The results in Table 7 indicate that enriching the rabbit cages affected the behavioral activities of the rabbits. The T3 and T4 rabbits showed lower standing and fear response values. We can attribute these results to the animal’s interaction with its surrounding objects and stress hormone levels (such as cortisol) in its blood. Enriching a rabbit’s environment provides the animal with the opportunity to express behavioral activity naturally, reaching behavioral enrichment [13,42]. These findings indicate that enriching an animal’s environment can reduce stress and improve welfare. Furthermore, stimulating tools, such as plastic-colored balls and mirrors, work as a distraction from the feeling of separation from a rabbit’s mother after weaning, can reduce physiological stress, can allow growing rabbits to move up/down, and can increase explorative behavior, while maintaining health and production statuses. Mirrors, for example, are known to add an additional dimension to the environment and increase the space in which the animal is located. Mirror self-recognition is a unique experimental window into an animal’s mind, because it is frequently seen as a marker of self-awareness [43]. In the same manner, Mastellone et al. [44] found that the use of mirrors may represent a low-cost efficient object for stimulating the manifestation of natural behaviors in rabbits bred in small groups in a free-range system. The rabbits raised in mirrored cages with visual and olfactory contact expressed much higher levels of crucial natural activities, such as olfactory investigations and allo-grooming activities, than other groups. These results are also consistent with Musco et al. [14], who also reported that mirrors can represent a valid approach for enhancing the well-being and productive performance of rabbits. 

## 5. Conclusions

Based on our knowledge, this research is the first to examine the impact of using a rubber floor, plastic-colored balls, and a mirror, in different environmentally enriched cages, on the physiological status, productive performance (including carcass quality), behavior, and welfare of growing V-line rabbits. According to the current findings, enriching rabbit cages with plastic-colored balls and mirrors positively affects rabbits’ productivity, carcass quality, behavior, and welfare. It also decreases the rabbits’ fear response and enhances the rabbits’ health and immunity. Successful enrichment strategies can provide direct and indirect benefits for rabbit behavior, health, productivity, welfare, and farm management. Therefore, it is recommended to apply such enrichment tools to rabbit cages, in addition to conducting further studies on employing combined environmental enrichments for better rabbit husbandry. 

## Figures and Tables

**Table 1:** Behaviors evaluated during the experiment.

Behavior	Description
Walking	Time rabbits spent walking on the cage floor.
Running	Time spent in running on the cage floor.
Standing	Time rabbits spent standing and immobilized on the floor.
Resting	Time spent in lying down on the cage floor without any activity.
Eating	Time spent in eating from feeders.
Drinking	Time spent in drinking from nipples.
Smelling	Time spent in sniffing and exploring around.
Self-grooming	Time spent in licking and cleaning the body.
Urination	Number of times rabbits urinated.
Fighting	Time rabbits spent fighting with each other.
Fear response	Time rabbits spent expressing emotional responses when confronted with surrounding threats.

**Table 2:** The effect of different cage enrichments on the productive performance of growing rabbits (mean ± SE).

Treatments	T1 (Control)	T2 (Rubber Floor)	T3 (Colored Balls)	T4 (Mirror)	*p* Value
Traits					
Initial BW (g)	528.14 ± 5.38	527.30 ± 4.98	527.91 ± 5.14	529.02 ± 4.89	0.169
Final BW (g)	1908.28 ^c^ ± 8.77	1915.84 ^c^ ± 9.31	1987.88 ^a^ ± 9.29	1968.37 ^b^ ± 9.08	0.001
BWG (g)	1381.92 ^c^ ± 6.81	1385.84 ^c^ ± 6.39	1462.35 ^a^ ± 6.44	1440.09 ^b^ ± 6.13	0.001
FI (g)	4021.02 ± 1.19	4023.31 ± 1.10	4045.11 ± 1.18	4039.27 ± 1.19	0.198
FCR	2.93 ^a^ ± 1.18	2.91 ^a^ ± 1.15	2.78 ^b^ ± 1.19	2.81 ^b^ ± 1.17	0.035
Mortality (%)	9.52 ± 0.13	9.53 ± 0.14	9.52 ± 0.12	9.52 ± 0.11	0.288

^a,b,c^ Means having different letters in the same row are significantly different (*p* ≤ 0.05). Initial BW = initial body weight at the 4th week of age. Final BW = final body weight at the 10th week of age. BWG = body weight gain from 4 to 10 weeks. FI = feed intake from 4 to 10 weeks. FCR = feed conversion rate from 4 to 10 weeks.

**Table 3:** The effects of different cage enrichments on the blood biochemical parameters of rabbits (mean ± SE).

Treatments	T1 (Control)	T2 (Rubber Floor)	T3 (Colored Balls)	T4 (Mirror)	*p* Value
Parameters					
Total protein (g/dL)	5.58 ^b^ ± 0.11	5.73 ^b^ ± 0.09	6.18 ^a^ ± 0.14	6.15 ^a^ ± 0.05	0.041
Albumin (g/dL)	3.99 ^b^ ± 0.04	3.89 ^b^ ± 0.12	4.15 ^a^ ± 0.05	3.96 ^b^ ± 0.12	0.035
Globulin (g/dL)	1.59 ^c^ ± 0.09	1.84 ^b^ ± 0.11	2.03 ^a^ ± 0.15	2.19 ^a^ ± 0.15	0.022
Alb/Glob ratio	2.51 ^a^ ± 0.14	2.11 ^b^ ± 1.10	2.05 ^b^ ± 0.22	1.83 ^c^ ± 0.18	0.008
Glucose (mg/dL)	141.86 ^b^ ± 5.05	135.71 ^c^ ± 4.05	152.80 ^a^ ± 3.09	149.85 ^a^ ± 3.95	0.005
Cholesterol (mg/dL)	79.20 ^c^ ± 4.13	64.62 ^d^ ± 2.10	84.40 ^b^ ± 3.31	93.62 ^a^ ± 2.32	0.001
Triglycerides (mg/dL)	66.60 ^b^ ± 4.36	61.25 ^b^ ± 2.69	64.03 ^b^ ± 2.17	75.80 ^a^ ± 2.04	0.003
HDL (mg/dL)	37.01 ^b^ ± 1.63	30.20 ^c^ ± 1.37	38.00 ^b^ ± 2.01	42.01 ^a^ ± 2.90	0.030
LDL (mg/dL)	28.89 ± 2.43	27.97 ± 1.71	29.49 ± 2.23	30.04 ± 1.18	0.062
AST (U/L)	42.41 ^b^ ± 1.60	43.80 ^b^ ± 1.08	54.60 ^a^ ± 1.20	55.20 ^a^ ± 1.37	0.001
ALT (U/L)	36.75 ^b^ ± 1.97	40.20 ^a^ ± 1.13	39.86 ^a^ ± 0.99	41.25 ^a^ ± 2.67	0.003
Creatinine (mg/dL)	0.74 ^b^ ± 0.02	0.78 ^a^ ± 0.03	0.77 ^a^ ± 0.04	0.78 ^a^ ± 0.02	0.001
IgG (mg/mL)	13.25 ^b^ ± 0.73	13.46 ^b^ ± 0.55	15.34 ^a^ ± 0.47	14.42 ^a^ ± 0.99	0.003
IgM (mg/mL)	6.72 ^c^ ± 0.35	6.66 ^c^ ± 0.41	8.18 ^a^ ± 0.54	7.77 ^b^ ± 0.17	0.001
T_3_ (ng/dL)	0.83 ^b^ ± 0.01	0.79 ^c^ ± 0.01	0.91 ^a^ ± 0.02	0.86 ^b^ ± 0.03	0.001
Corticosterone (ng/mL)	14.41 ^a^ ± 0.26	15.01 ^a^ ± 1.05	12.58 ^b^ ± 0.21	12.05 ^b^ ± 0.21	0.004

^a,b,c,d^ Means having different letters in the same row are significantly different (*p* ≤ 0.05). Alb/Glob = albumin/globulin. HDL = high-density lipoprotein. LDL = low-density lipoprotein. AST = aspartate aminotransferase. ALT = alanine transaminase. IgG = immunoglobulin G. IgM = immunoglobulin M. T_3_ = triiodothyronine.

**Table 4:** The effects of different cage enrichments on the blood hematological parameters of rabbits (mean ± SE).

Treatments	T1 (Control)	T2 (Rubber Floor)	T3 (Colored Balls)	T4 (Mirror)	*p* Value
Parameters					
RBC_S_ (10^6^/mm^3^)	4.60 ^b^ ± 0.09	4.52 ^b^ ± 0.05	4.73 ^a^ ± 0.04	4.75 ^a^ ± 0.06	0.014
Hb (g/dL)	10.75 ^b^ ± 0.11	10.86 ^b^ ± 0.10	11.63 ^a^ ± 0.10	11.46 ^a^ ± 0.12	0.045
Hematocrit (%)	33.56 ^c^ ± 1.23	35.02 ^b^ ± 1.35	35.86 ^a^ ± 1.20	36.01 ^a^ ± 1.33	0.041
WBC_S_ (10^3^/mm^3^)	9.29 ^a^ ± 0.23	9.57 ^a^ ± 0.24	8.80 ^b^ ± 0.25	8.82 ^b^ ± 0.27	0.028
Lymphocyte (%)	68.39 ± 3.08	69.74 ± 2.18	68.21 ± 2.13	68.47 ± 2.65	0.075
Neutrophil (%)	23.14 ± 2.82	23.29 ± 1.70	23.10 ± 1.90	22.91 ± 1.66	0.085
Monocyte (%)	4.96 ± 0.40	5.01 ± 0.35	4.72 ± 0.32	4.83 ± 0.37	0.062

^a,b,c^ Means having different letters in the same row are significantly different (*p* ≤ 0.05). RBCs = red blood cells. Hb = hemoglobin. WBC_S_ = white blood cells.

**Table 5:** The effects of different cage enrichments on the carcass characteristics and relative weight of internal organs in rabbits (mean ± SE).

Treatments	T1 (Control)	T2 (Rubber Floor)	T3 (Colored Balls)	T4 (Mirror)	*p* Value
Parameters					
Carcass weight (g)	1061.39 ^b^ ± 3.78	1068.45 ^b^ ± 4.01	1155.56 ^a^ ± 3.55	1140.28 ^a^ ± 3.87	0.012
Dressing (%)	55.62 ^b^ ± 0.41	55.77 ^b^ ± 0.39	58.13 ^a^ ± 0.34	57.93 ^a^ ± 0.48	0.003
Liver (%)	3.86 ± 0.10	3.88 ± 0.10	3.91 ± 0.11	3.89 ± 0.10	0.088
Heart (%)	0.36 ± 0.02	0.37 ± 0.01	0.42 ± 0.02	0.39 ± 0.02	0.169
Kidney (%)	0.75 ± 0.02	0.76 ± 0.01	0.79 ± 0.02	0.77 ± 0.01	0.098
Spleen (%)	0.09 ± 0.01	0.09 ± 0.01	0.11 ± 0.01	0.10 ± 0.01	0.060
Intestine length (cm)	384 ^c^ ± 28.79	376 ^d^ ± 29.11	396 ^a^ ± 29.43	390 ^b^ ± 29.21	0.008

^a,b,c,d^ Means having different letters in the same row are significantly different (*p* ≤ 0.05).

**Table 6:** The effects of different cage enrichments on the cecal bacteria counts of growing rabbits (mean ± SE).

Treatments	T1 (Control)	T2 (Rubber Floor)	T3 (Colored Balls)	T4 (Mirror)	*p* Value
Bacteria Species					
*Lactobacillus* spp. (log_10_ CFU/g)	6.99 ± 1.14	7.03 ± 1.30	6.98 ± 1.17	6.98 ± 1.36	0.085
*Salmonella* spp. (log_10_ CFU/g)	3.48 ± 1.47	3.51 ± 1.61	3.50 ± 1.38	3.49 ± 1.50	0.098
*Escherichia coli* (log_10_ CFU/g)	5.69 ± 1.25	5.70 ± 1.36	5.69 ± 1.21	5.68 ± 1.29	0.091

**Table 7:** The effects of different cage enrichments on the behavioral activities of rabbits.

Behavioral Activities	T1 (Control)	T2 (Rubber Floor)	T3 (Colored Balls)	T4 (Mirror)
Walking	0	+1	0	0
Running	0	0	0	0
Standing	0	0	−1	−1
Resting	0	−1	+1	+1
Eating	0	0	0	0
Drinking	0	0	0	0
Smelling	0	0	+1	0
Self-grooming	+1	0	0	0
Urination	0	0	0	0
Fighting	0	0	0	0
Fear response	0	0	−1	−1

−1 = mild, 0 = normal, +1 = severe.

## Data Availability

The supplementary data can be available from the corresponding author upon reasonable request.
